# A Perplexing Case Highlighting the Diagnostic Conundrum of Miliary Tuberculosis Mimicking Sarcoidosis and Progressing Into Hemophagocytic Lymphohistiocytosis

**DOI:** 10.7759/cureus.78636

**Published:** 2025-02-06

**Authors:** Weiying Li, Prachi Mann, Ivonne De La Hoz, Alexa Constantakos, Dwayne Gordon, George Everett, Edward Maharam

**Affiliations:** 1 Internal Medicine, AdventHealth, Orlando, USA

**Keywords:** hemophagocytic lymphohistiocytosis, hlh, miliary tb, sarcoidosis, tuberculosis

## Abstract

Establishing the diagnosis of miliary tuberculosis (TB) can be challenging due to the heterogeneous clinical presentations and low sensitivity of diagnostic tests. Miliary TB shares overlapping clinical, radiological, and histopathological features with other chronic granulomatous diseases, such as sarcoidosis, often posing a significant diagnostic challenge for clinicians. A 36-year-old male from Haiti presented with a four-month history of recurrent fever, dry cough, night sweats, and weight loss. Chest imaging revealed innumerable widespread miliary nodules throughout the lungs bilaterally, raising a high clinical suspicion for miliary TB. The work-up for bacterial, fungal, and viral infection was negative, and there was no evidence of malignancy. Surprisingly, extensive TB testing yielded negative results. The interferon-gamma release assay (QuantiFERON TB Gold Plus®), *Mycobacterium tuberculosis* (MTB) complex polymerase chain reaction (PCR), and repetitive sputum cultures for acid-fast bacilli (AFB) were all negative. A lung biopsy was performed due to an unexpectedly negative tuberculosis work-up and revealed non-necrotizing granulomatous inflammation, with no AFB identified on bronchoalveolar lavage (BAL) or histopathological staining.

Additionally, the next-generation sequencing technique was conducted using microbial cell-free DNA and was negative for tuberculosis or any other pathogen. Therefore, sarcoidosis was considered the most likely diagnosis based on the exclusion of infectious etiologies. The patient was started on high-dose steroids. However, the patient failed to respond clinically and developed worsening transaminitis, uptrending ferritin, and pancytopenia. His H-score was as high as 256 points, which suggested the probability of hemophagocytic lymphohistiocytosis (HLH) was as high as 99%. A bone marrow biopsy revealed multiple small foci of noncaseating granulomatous inflammation with hemophagocytic cells. The patient was started on etoposide steroids and empiric broad-spectrum antibiotics. Despite aggressive management, the patient’s condition rapidly deteriorated as he developed acute hypoxic respiratory failure requiring mechanical ventilation, refractory shock, and multi-organ failure.

The infection screen was repeated due to the worsening clinical status. To everybody’s surprise, the repeated next-generation sequencing on hospital day 26 detected MTB complex. This was confirmed with positive AFB staining, MTB complex PCR, and AFB cultures on BAL samples. A four-drug anti-TB regimen was promptly commenced. However, the patient's condition continued to deteriorate rapidly, and the patient expired one week later. This complex case raised several diagnostic and therapeutic dilemmas. First of all, the optimal treatment approach remains unclear, considering the risk of infection secondary to immunosuppressants for HLH might outweigh the benefits. It also highlights the diagnostic limitations of TB testing and the overreliance on diagnostic tests that should be avoided, especially when there is a high pretest probability of miliary TB. Early initiation of empirical anti-TB therapy should be considered to improve outcomes, considering the mortality of miliary TB is extremely high.

## Introduction

Establishing the diagnosis of miliary tuberculosis (TB) has been challenging due to the limited yield of diagnostic tests. Other conditions, such as sarcoidosis or disseminated fungal infection, share very similar clinical presentations, and differentiation can be challenging, especially in the context of negative TB tests. Negative initial TB tests should not completely exclude the possibility of miliary TB, especially with very strong clinical suspicion [1]. We present a perplexing case of miliary TB that was initially thought to be sarcoidosis and then progressed into hemophagocytic lymphohistiocytosis (HLH). 

## Case presentation

A 36-year-old male from Haiti with no significant past medical history presented to the emergency department due to fever, dry cough, night sweats, and unintentional weight loss for four months. He was tachycardiac and febrile on admission, with a temperature of 39°Celcius (102.2°F) and a heart rate of 115 per minute. Initial laboratory studies were only remarkable for mild normocytic anemia and elevated inflammatory markers. HIV test was negative (Table 1).

**Table 1:** Laboratory trends. WBS: white blood cells, ALT: alanine aminotransaminase; AST: aspartate aminotransaminase.

Items	Day 1	Day 18	Reference range
Hemoglobin (g/dL)	9.9	6.8	11.4-14.7
Platelet (10^3^/μL)	227	40	139-36
WBC count (10^3^/μL)	4.5	0.03	4.4-10.5
Absolute neutrophil (10^3^/μL)	2.9	0	1.5-7.5
Ferritin (ng/mL)	1364	7821	24-336
AST (U/L)	63	335	5-46
ALT (U/L)	40	325	4-51

**Figure 1:**
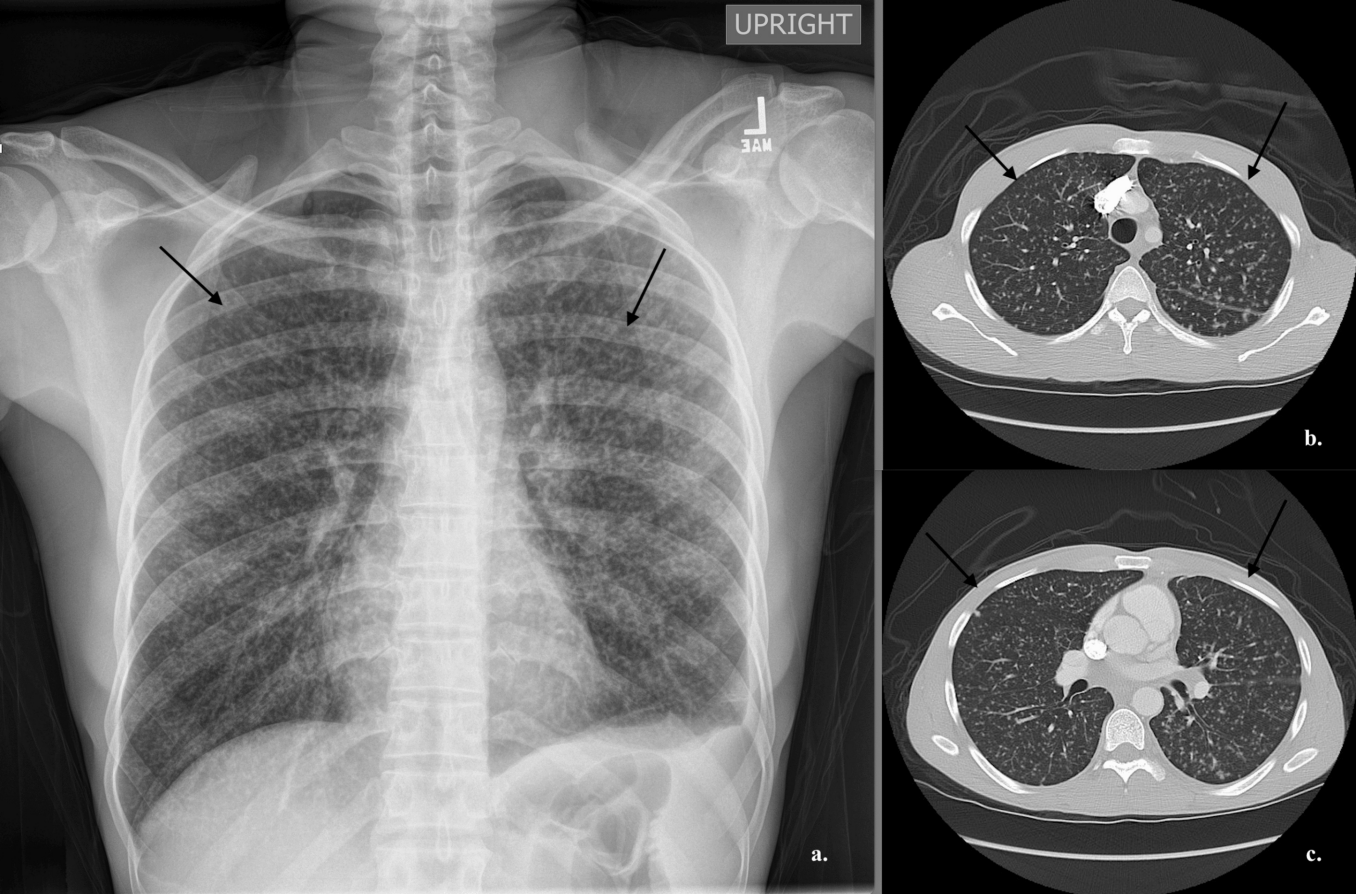
Chest X-ray and CT of chest. Both chest X-ray (a) and computer tomography (CT) chest (b and c) showed innumerable widespread bilateral miliary nodules suggestive of miliary tuberculosis.

Initial chest X-ray showed innumerable widespread bilateral miliary nodules suggestive of miliary tuberculosis, and computed tomography (CT) of the abdomen revealed moderate splenomegaly (Figure 1).

**Figure 2:**
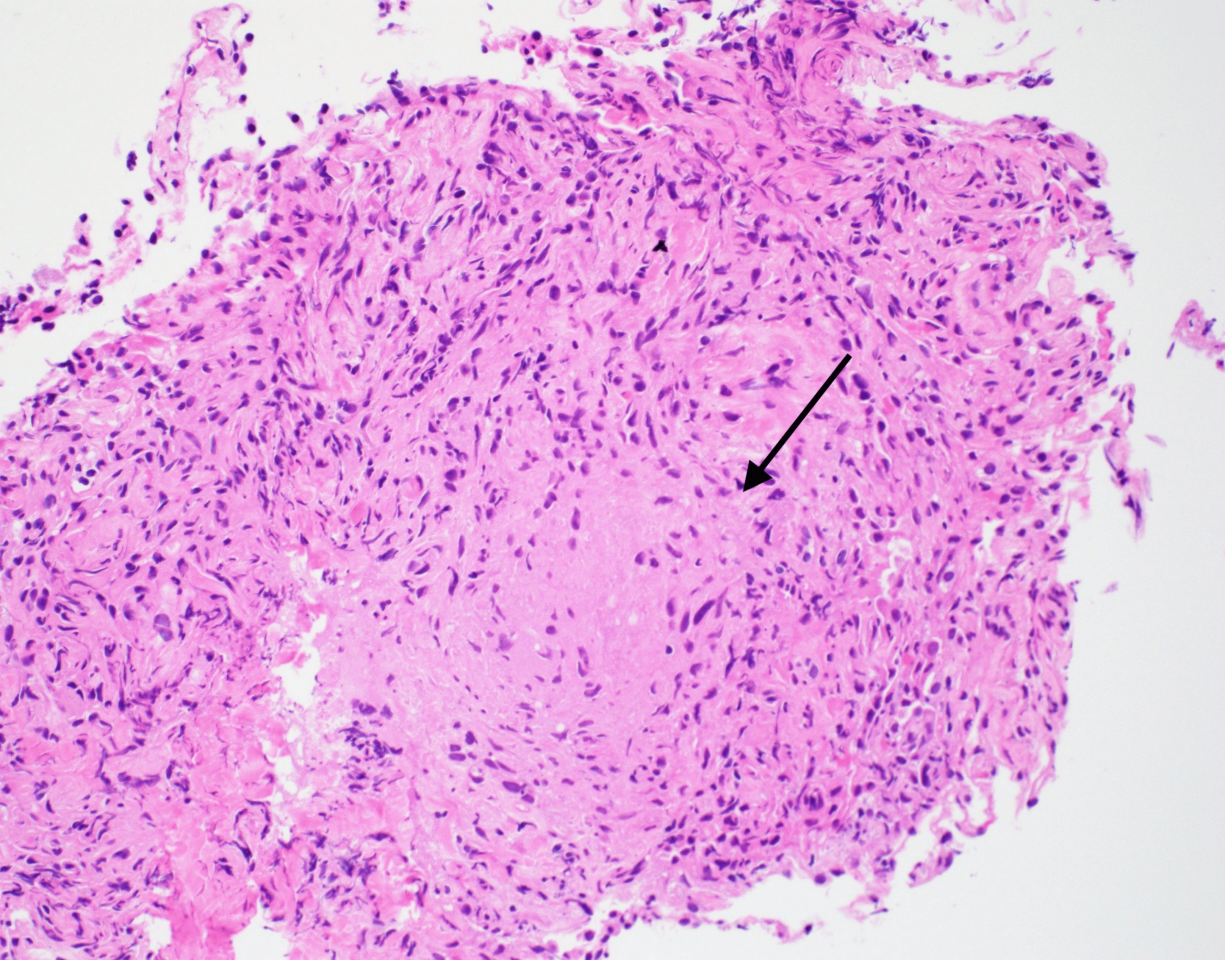
Non-caseating granuloma in lung biopsy.

The initial workup was focused on confirming the diagnosis of tuberculosis, and the infectious disease team was about to start anti-tuberculosis treatment once we detected the pathogen. Unexpectedly, the interferon-gamma release assay (QuantiFERON TB Gold Plus®)was negative. Further work-up, including acid-fast bacilli (AFB) smear, *Mycobacterium tuberculosis* (MTB) complex PCR, and mycobacterial cultures, were all negative. Furthermore, bronchoalveolar lavage (BAL) was collected, but negative AFB smear and mycobacterial cultures were reported. In light of unexpected negative results from repeated sputum specimens, a transbronchial lung biopsy was performed and showed non-necrotizing granulomatous inflammation; however, AFB staining on lung biopsy tissue was negative (Figure 2). 

Concurrent diagnostic methods using next-generation sequencing techniques (MicroGenDX® and Karius®) were also negative for MTB. With such an extensive diagnostic effort combined with advanced molecular testing all pointing away from tuberculosis, it appeared that tuberculosis and other infectious causes had been reasonably excluded. At this stage, an alternative diagnosis, sarcoidosis, started emerging based on the finding of non-caseating granuloma on biopsy and the diagnosis of exclusion. Therefore, the patient was started on a high-dose steroid after every possible test had excluded the possibility of tuberculosis. However, the patient continued having daily fever and dry cough without showing any significant improvement.

**Figure 3:**
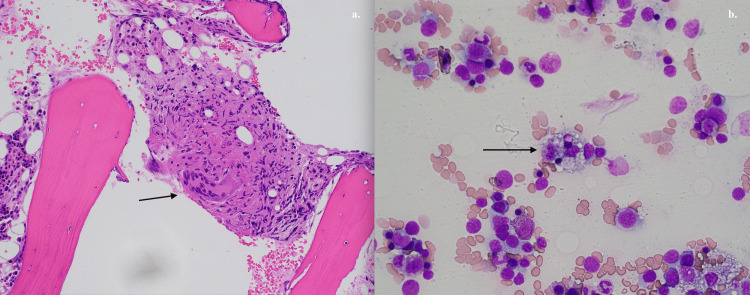
Bone marrow biopsy and aspiration. a.  Bone marrow biopsy showing non-caseating granuloma. b. Bone marrow aspiration showing hemophagocytic cells.

Meanwhile, lab monitoring showed worsening pancytopenia and a significant up-trending of liver enzymes and ferritin, which raised the concern of hemophagocytic lymphohistiocytosis (HLH). The calculation of H-Score was 256 points, which suggested a 99% probability of HLH. Furthermore, the more specific markers, including soluble interleukin 2 receptors (7,593.1 pg/mL, reference range 175.3-858.2 pg/mL) and CXCL9 (52,784 pg/mL, reference range<647 pg/mL), were both markedly elevated. A bone marrow biopsy revealed multiple small foci of noncaseating granulomatous inflammation with occasional hemophagocytic cells (Figure 3).

**Figure 4:**
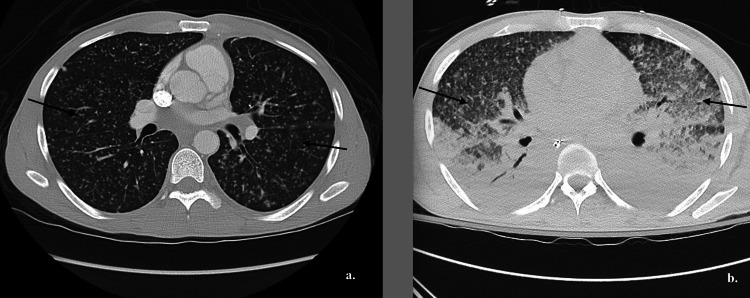
Evolution of CT chest. (a) CT chest on day 1, showing diffuse bilateral miliary nodules filling the entirety of both lungs, (b) CT chest on day 30, note the diffuse bilateral reticulonodular, flocculent, and consolidative airspace opacities.

After a thorough discussion with a multidisciplinary team, the patient was started on HLH treatment with high-dose steroids and etoposide based on the HLH-2004 protocol [2]. On hospital day 19, the patient developed acute hypoxic respiratory failure, ultimately requiring endotracheal intubation and profound shock that required multiple vasopressors. Broad-spectrum antibiotics and antifungals were started empirically due to clinical deterioration. A CT chest angiogram excluded the pulmonary embolism but revealed new airspace opacities throughout bilateral lungs, which progressively worsened compared to day 1 (Figure 4).

**Figure 5:**
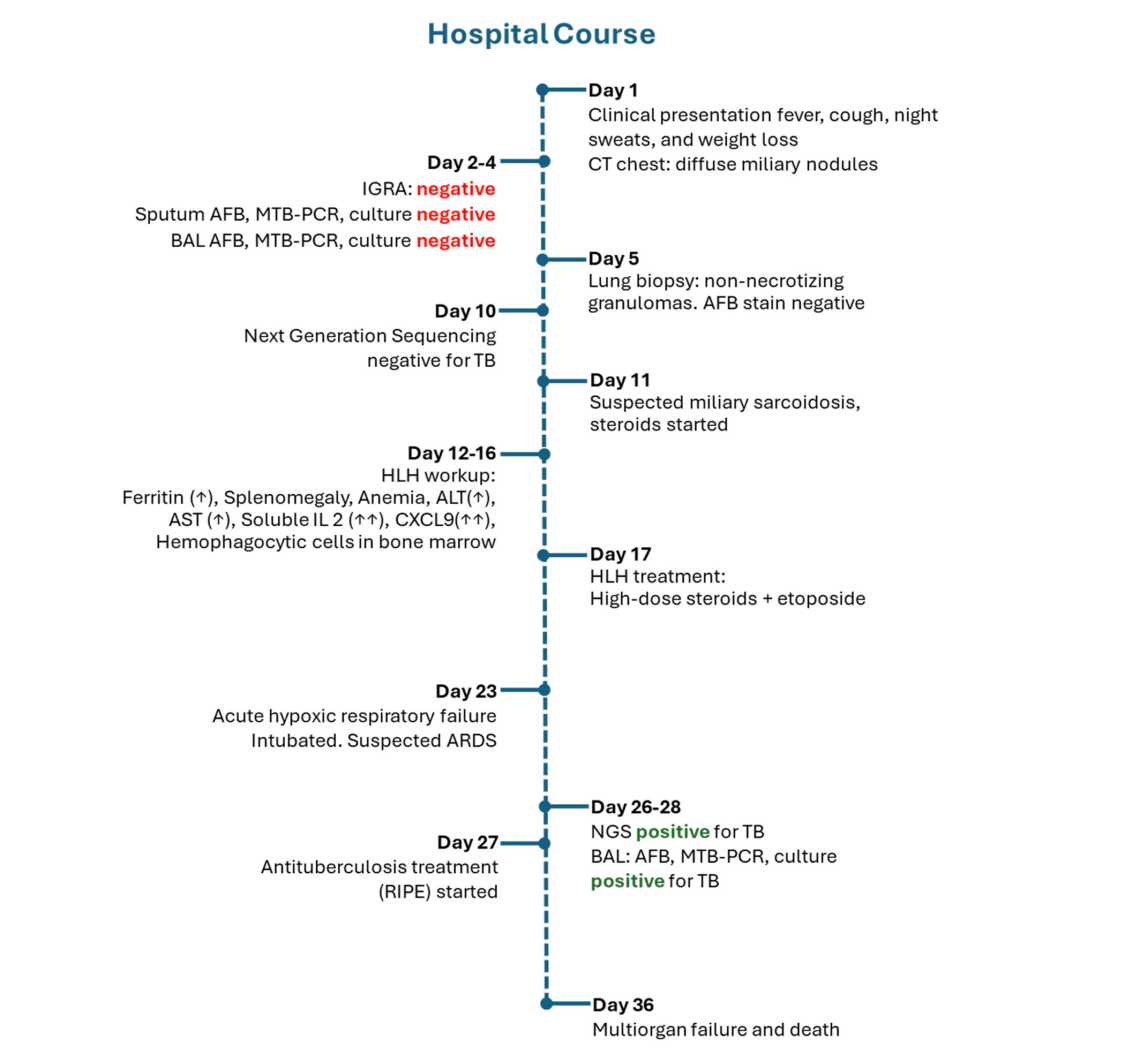
Timeline of events during hospitalization. IGRA: interferon-gamma release assay; BAL: bronchoalveolar lavage; AFB: acid-fast bacilli; MTB: *Mycobacterium tuberculosis*; PCR: polymerase chain reaction; NGS: next-generation sequencing; HLH: hemophagocytic lymphohistiocytosis; IL: interleukin CXCL9; ALT: alanine aminotransferase; AST: aspartate aminotransferase; RIPE: rifampin, isoniazid, pyrazinamide, ethambutol.

These radiographic findings raised concern for the development of acute respiratory distress syndrome (ARDS). Next-generation sequencing tests (Karius® and MicroGenDX®) were repeated, and unexpectedly, M*ycobacterium tuberculosis *complex was detected on both tests. A second bronchoalveolar lavage was repeated, and AFB staining and MTB complex PCR were positive within hours. An anti-tuberculosis regimen comprised of rifampin, isoniazid, pyrazinamide, and ethambutol (RIFE) was initiated while the chemotherapy for HLH treatment was withheld. Further testing with mycobacterial cultures also confirmed the presence of *Mycobacterium tuberculosis*. Unfortunately, the patient rapidly declined and developed multiorgan failure. He expired after a prolonged and complicated hospital course despite maximum support. The timeline of events is illustrated in Figure 5.

## Discussion

Of all the questions and uncertainties about this case, the biggest question is; “In which stage of the clinical course did the patient develop TB?” There are two possibilities: one is that the patient initially presented with miliary TB, as supported by epidemiological exposure, textbook-like clinical symptoms, and radiological findings despite initial negative TB tests; another possibility is that the patient developed re-activation of TB after receiving high dose steroid and chemotherapy for HLH, as it is not uncommon that TB could develop or be reactivated in patients who become immunocompromised after receiving biologic agent or chemotherapy [3]. We think it is more likely that the patient had tuberculosis at the very beginning, but we do not have a definite answer to this question because the family declined the autopsy. We have learned so much from this case, and we think it is worthwhile to share this so we all can learn from it. Our patient immigrated from Haiti, where the prevalence of latent TB is reported as high as 54.8% [4]. Based on the report from the World Health Organization (WHO), the estimated incidence of TB in Haiti is 176 new cases per 100,000 people annually. On admission, diagnostic certainty for miliary TB was very high, given epidemiological risk factors, typical clinical symptoms, and radiological findings. Only when laboratory studies came back negative for TB did the diagnostic certainty fall. 

Limitations of diagnostic tests in miliary TB

Miliary tuberculosis is a disseminated form of tuberculosis characterized by widespread granulomatous lesions in multiple organs. It is named miliary because the tiny lesions that form in the affected organs resemble millet seeds on imaging, as manifested in the chest X-ray of this case. Although QuantiFERON-TB Gold Plus (QFT-TB) is a useful tool that has 67-89% sensitivity and 98.1% specificity for active and latent TB [1,5], the evidence for the use of QFT-TB test in miliary tuberculosis is inadequate. It is important to point out that the negative predictive value of QFT-TB is much lower for populations from endemic areas where the prevalence of latent TB is very high. One study reported the diagnostic yield of QFT-TB in miliary TB could be as low as 68%, which may correlate with a decrease in the production of interferon (IFN)-γ resulting from immunosuppression and lymphopenia [6], therefore a negative QFT-TB test does not rule out miliary TB [7]. The diagnostic sensitivity of AFB staining does not exceed 50% [8] and can be as low as 37% in military TB [9]. Studies have shown that bronchoalveolar lavage (BAL) can increase the positive rate, but the diagnostic yield still varies widely, with a positive rate of 31.6-47% with BAL smear and 30.5%-78% with BAL PCR in pulmonary TB [10,11].

The diagnostic yield of a transbronchial biopsy for miliary tuberculosis is between 62.5% and 76%, even though one study from India reported only 1 in 21 cases was found with positive AFB stain in lung biopsy [12]. Alone, each of these diagnostic methods has relatively low diagnostic sensitivity; however, the diagnostic yield can be increased when used in combination and when biopsy samples can be obtained from as many sites as possible [13-16]. Advanced diagnostic tools such as next-generation sequencing (NGS) using cell-free DNA are emerging methods in the field of infectious diseases. A meta-analysis included 18 studies that reported pooled sensitivity and specificity of 48-68% and 91-99%, respectively, suggesting a relatively low sensitivity but high specificity of using cell-free DNA in diagnosing tuberculosis [17]. Karius cfDNA has an advantage in detecting slow-growing pathogens such as tuberculosis and has the potential to expedite the diagnosis and treatment initiation and decrease the need for invasive diagnostics [18]. In 2023, WHO released a communication supporting the use of target NGS to detect drug resistance after TB diagnosis to guide clinical decisions for drug-resistant TB treatment. A retrospective study showed that the sensitivity of NGS was 78.3% when confirming the diagnosis of pulmonary TB, demonstrating a higher detection capability in lung biopsy tissue compared with BAL [19]. Multiple cases have reported initial negative QFT, AFB staining, and MTB PCR but were later proven to be positive for TB in re-assessment, with subsequent patient improvement with anti-TB treatment [20]. Some cases failed to establish the diagnosis of TB with all tests, including AFB stain, MTB PCR, and culture; however, the autopsy revealed miliary TB after the patient expired from untreated TB [21,22]. This suggests the importance of a prudent interpretation of test results considering their imperfect performance, especially in the case of disseminated or miliary TB [23] and in immunocompromised patients [24].

Was there sarcoidosis?

Sarcoidosis is a multisystem granulomatous disorder with unknown pathogenesis. Some theories propose that granulomatous inflammation might be triggered by environmental exposures or infectious agents. There is significant overlap in clinical manifestations, radiological findings, and pathological features between sarcoidosis and TB; therefore, excluding TB is a mandatory step before establishing the diagnosis of sarcoidosis [25]. Sarcoidosis can also be considered when a remarkable clinical improvement cannot be achieved with standard anti-TB drugs administered at full dosage [26]. In our case, stage III sarcoidosis was diagnosed based on nodular pulmonary lesions, biopsy-proven non-caseating granuloma, and repetitive tests negative for TB and other infectious organisms such as HIV, *cryptococcus, aspergillus, histoplasmosis, PJP, brucella*, John Cunningham virus (JC) virus. Typical radiographic findings of sarcoidosis feature bilateral hilar adenopathy and reticular opacities that are distributed along the broncho-vascular bundle and predominantly involve the upper middle lobe. Miliary sarcoidosis, although rare, may present with miliary pattern of nodules that is hard to distinguish from that of miliary TB [27]. Typically, the classical pathological feature of TB is caseating granuloma compared to non-caseating granuloma found in sarcoidosis. However, the distinction between caseating and noncaseating is not absolute since it depends on a subjective judgment from clinicians [28]. Studies have shown that noncaseating granulomas may be present with TB [29], and sarcoidosis granulomas have necrotic features in up to one-third of cases [30,31]. Historically, significant efforts have been made to study the association between TB and sarcoidosis, as these two diseases are deeply intertwined. The current consensus recommends excluding TB before establishing the diagnosis of sarcoidosis. However, a case has proposed the coexistence of TB and sarcoidosis in which the patient did not fully respond to initial anti-TB treatment but fully recovered after adding steroids to the treatment [32,33]. Interestingly, some researchers even consider sarcoidosis and TB as two examples of the same disease process [34]. Examples of cases reporting TB preceding the development of sarcoidosis, whereas another case reported TB as a complication of treatment in sarcoidosis [35]. Recent molecular and immunological studies suggest mycobacterial antigens are the inciting agents in a proportion of sarcoidosis patients. Therefore, the possibility has been raised that MTB might be the cause of sarcoidosis and may participate in the pathogenesis of sarcoidosis [36]. More studies are warranted to elucidate etiology and pathogenesis further. Questions remain regarding the optimal approach to cases where TB is highly suspected due to clinical reasoning despite negative tests, and sarcoidosis remains a differential diagnosis. Considering the significantly limited diagnostic value of TB tests in miliary TB, we recommend empirically starting anti-TB treatment if clinically suspected; then, steroids could be implemented if there is an inadequate clinical response to the initial anti-TB treatment. An alternative option is to start both anti-TB and steroids as initial treatment in perplexing cases like this one.

Does miliary TB trigger hemophagocytic lymphohistiocytosis?

In this case, the patient developed hemophagocytic lymphohistiocytosis (HLH), which was diagnosed based on fever, cytopenia, the presence of hemophagocytosis in bone marrow, ferritin higher than 500 ng/mL, and elevated soluble IL-2 receptor. There have been rare cases of sarcoidosis triggering HLH, and numerous reports show that miliary TB can serve as a culprit to HLH as well [37]. These studies emphasize the importance of correcting the primary cause and initiating anti-TB treatment early rather than treating HLH alone. A retrospective study identified 63 cases of TB-associated HLH, finding that TB complicated by HLH had a mortality as high as 49%, and delaying anti-TB treatment is associated with poor survival. Another study reviewed 116 cases of TB-associated HLH; it revealed that in all cases where anti-TB treatment was not given, the patients succumbed to their illness. Survival improved from 55% to 72% by adding immunomodulating treatment to anti-TB treatment [38,39]. Based on the low-quality evidence from the case series, it seems adding IVIG and plasma exchange might improve patients’ outcomes [40]. However, the utility of immunosuppressive/immunomodulator therapy lacks general consensus among physicians and warrants further study [41].

On the other hand, another study suggested antituberculosis treatment was associated with improved survival, independent of steroid therapy. It concluded that starting treatment for underlying TB disease was the only modifiable factor associated with lower mortality [42,43]. In summary, the optimal treatment for miliary TB with HLH is unclear, and additional studies are needed to guide treatment in such challenging clinical scenarios.

## Conclusions

Overall, diagnostics tests available clinically have significant limitations and low diagnostic yield in detecting miliary TB, which poses major challenges to its diagnosis, therefore we should not overly rely on tests when it comes to diagnosing miliary TB. In clinical scenarios where TB is highly suspected despite negative TB tests, empirically starting anti-TB treatment is highly recommended. The mortality of miliary TB complicated by HLH is extremely high, and early initiation of TB treatment appears to be the only intervention with survival benefits.
